# Transcriptomic Changes in Internode Explants of Stinging Nettle during Callogenesis

**DOI:** 10.3390/ijms222212319

**Published:** 2021-11-15

**Authors:** Xuan Xu, Sylvain Legay, Roberto Berni, Jean-Francois Hausman, Gea Guerriero

**Affiliations:** Environmental Research and Innovation Department, Luxembourg Institute of Science and Technology, 5, Rue Bommel, Hautcharage, L-4940 Luxembourg, Luxembourg; xuan.xu@list.lu (X.X.); sylvain.legay@list.lu (S.L.); roberto.berni@list.lu (R.B.); jean-francois.hausman@list.lu (J.-F.H.)

**Keywords:** callogenesis, transcriptomics, stinging nettle, qPCR, plant growth regulators

## Abstract

Callogenesis, the process during which explants derived from differentiated plant tissues are subjected to a trans-differentiation step characterized by the proliferation of a mass of cells, is fundamental to indirect organogenesis and the establishment of cell suspension cultures. Therefore, understanding how callogenesis takes place is helpful to plant tissue culture, as well as to plant biotechnology and bioprocess engineering. The common herbaceous plant stinging nettle (*Urtica dioica* L.) is a species producing cellulosic fibres (the bast fibres) and a whole array of phytochemicals for pharmacological, nutraceutical and cosmeceutical use. Thus, it is of interest as a potential multi-purpose plant. In this study, callogenesis in internode explants of a nettle fibre clone (clone 13) was studied using RNA-Seq to understand which gene ontologies predominate at different time points. Callogenesis was induced with the plant growth regulators α-napthaleneacetic acid (NAA) and 6-benzyl aminopurine (BAP) after having determined their optimal concentrations. The process was studied over a period of 34 days, a time point at which a well-visible callus mass developed on the explants. The bioinformatic analysis of the transcriptomic dataset revealed specific gene ontologies characterizing each of the four time points investigated (0, 1, 10 and 34 days). The results show that, while the advanced stage of callogenesis is characterized by the iron deficiency response triggered by the high levels of reactive oxygen species accumulated by the proliferating cell mass, the intermediate and early phases are dominated by ontologies related to the immune response and cell wall loosening, respectively.

## 1. Introduction

Differentiated plant cells retain the ability to trans-differentiate to callus [1], i.e., lose their committed fate and re-enter the cell cycle, proliferate to produce a mass of cells known as callus and, under specific conditions and phytohormonal stimuli, regenerate tissues and whole organs [2]. This plasticity is at the base of many biotechnology-related applications, such as micropropagation, plant transformation and establishment of cell suspension cultures. Plant bioprocess engineering approaches are receiving much attention in the light of their use in industrially relevant applications, e.g., to produce phytochemicals with bioactivities of interest for the cosmetic and food industries [3,4], as well as for the biopharmaceutical industry [5].

Among the plants of interest for the production of phytochemicals, e.g., for medicinal and antimicrobial applications, as well as for use in dietary supplements [6] and cosmetics [7], there is the common herbaceous species *Urtica dioica* L., also known as stinging nettle. Among the phenolic compounds of medicinal interest in nettle, it is worth mentioning lignans, whose abundance was shown to differ according to the tissues, i.e., aerial parts vs. roots [8]. Besides the production of phytochemicals, nettle is valued as a source of cellulosic fibres, the bast fibres [9,10], which can be used in biocomposites [11] and textiles [12]. More recently, carbon nanosheets with interesting physico-chemical properties, namely, interconnectivity of pores, graphitization, surface area and pore width [13], were prepared from stems of stinging nettles, which diversifies the application opportunities of this weed.

Establishing plant cell suspension cultures allows valued compounds to be produced on large scale [14], without having to source wild plants (thereby contributing to preserve native populations), or use lands, with lower environmental impact and completely independently of the season [4]. Callogenesis is a key step for the establishment of plant cultures. Molecular studies on the model plant *Arabidopsis thaliana* have allowed researchers to understand the mechanisms involved in plant callogenesis; such mechanisms involve the activation of the pathway mediating lateral root formation [15,16,17].

To establish plant cell cultures, tissue explants are cultivated on specific media containing a balance of growth regulators, i.e., auxins and cytokinins; the proliferating callus masses appearing are then separated and transferred to new media for their further growth. Established plant calli, i.e., sub-cultured over several weeks and showing a stable growth, are subsequently transferred to liquid media in shake flasks, usually containing the same balance of plant growth regulators (PGRs) and induced to disaggregate and form a homogeneous suspension culture. Hence, it is evident that the process of callogenesis is, at the same time, crucial and limiting for plant cell culture technologies and bioprocesses.

In the present study, callogenesis was studied from a molecular angle in stinging nettle, a plant for which no prior data exist and an herbaceous species holding great (and still under-exploited) industrial interest as a source of bioactive phytochemicals. The approach adopted relies on high-throughput sequencing of cDNA libraries prepared from stem segments at different steps of callogenesis, i.e., at 0, 1, 10 and 34 days.

## 2. Results and Discussion

### 2.1. Identification of Optimal Concentrations of Plant Growth Regulators Promoting Callogenesis

As a first step towards establishing an optimal protocol for callogenesis, different concentrations of the PGRs α-napthaleneacetic acid (NAA) and 6-benzyl aminopurine (BAP) were tested. These PGRs belong to the auxin and cytokinin families, respectively, and were chosen since they are known to regulate callus formation through both cooperative and antagonistic interactions [18].

**Figure 1:**
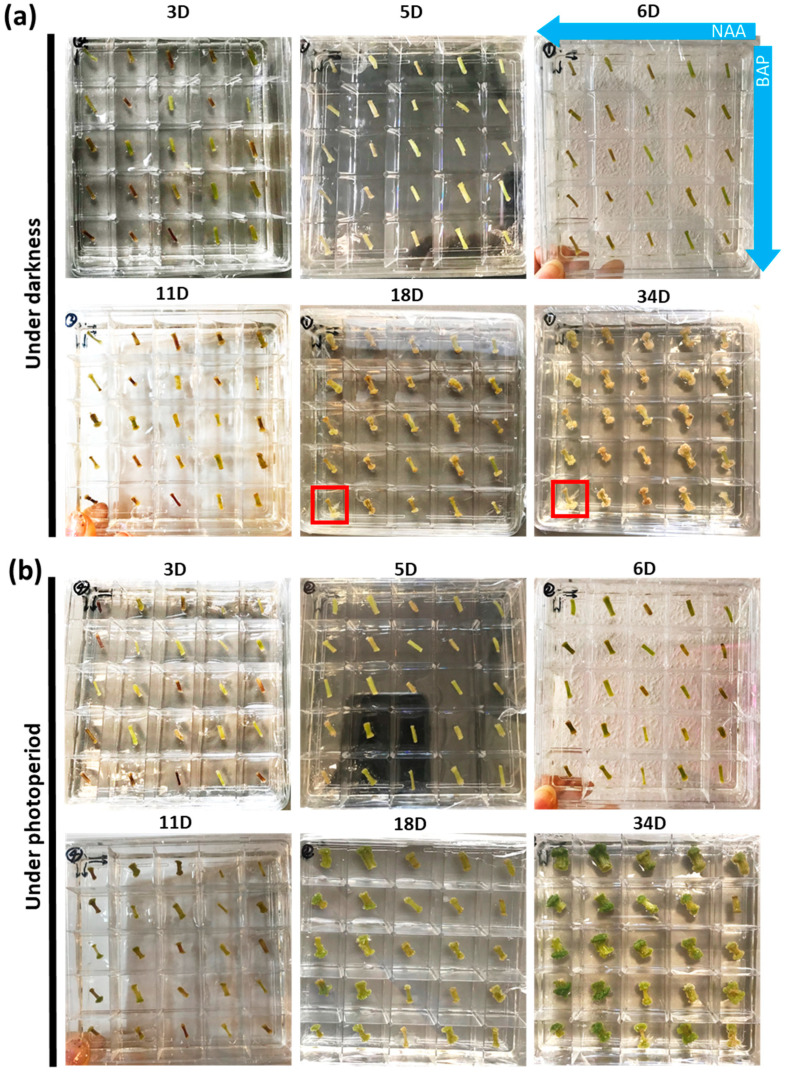
Pictures showing the effects of plant growth regulators at different concentrations and combinations on callogenesis of nettle explants at days 3, 5, 6, 11, 18 and 34 (D) in the dark (**a**) or photoperiod (**b**). The combination of different concentrations of NAA and BAP (i.e., 3, 1, 0.5, 0.1 and 0.01 mg/L) shown in Supplementary Figure S1 were applied for each condition. The blue arrows indicate the direction of the decreasing concentration of NAA and BAP. Explants with roots are highlighted with a red square.

Square Petri plates divided into 25 small compartments each containing a different combination of the PGRs (Supplementary Figure S1) were used. The test was carried out using two Petri dishes (i.e., 2 biological replicates) over a period of 34 days, both in darkness (Figure 1a) and photoperiod (Figure 1b). The time points taken for analysis were 3, 5, 6, 11, 18 and 34 days. As shown in Figure 1, calli formed at the extremities of the explants both in darkness and photoperiod. In darkness, roots appeared at the lowest concentrations of both NAA and BAP; at the highest concentration of NAA, calli appeared from day 11 onwards in darkness and photoperiod. At day 34, a well-developed creamy and compact green callus mass appeared in darkness and photoperiod, respectively, at the highest [NAA].

**Figure 2:**
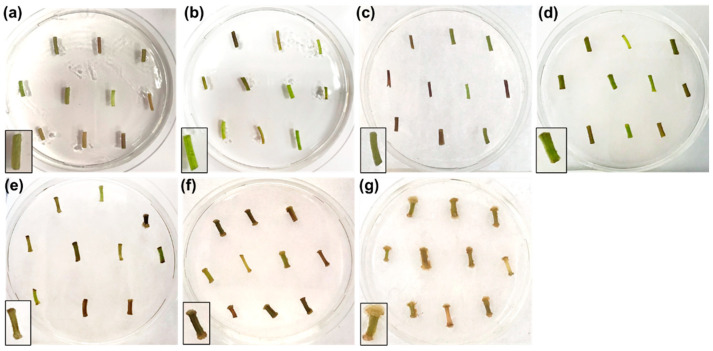
Pictures showing the effects of 3 mg/L NAA and 0.01 mg/L BAP on callogenesis of nettle explants at days 0 (**a**), 1 (**b**), 3 (**c**), 6 (**d**), 10 (**e**), 20 (**f**) and 34 (**g**) in the dark. Insets: details on the morphology of the internode explants at different time points.

Since a creamy colored callus developed in darkness and considering that dark cultivation of cell suspension cultures is economically more convenient than adopting photoperiod, a subsequent experiment was established in which the callogenesis of nettle internodes was followed at 0, 1, 3, 6, 10, 20 and 34 days in the dark using the concentrations of 3 mg/L for NAA and 0.01 mg/L for BAP (Figure 2). These concentrations were very effective in inducing the development of a creamy mass of callus (Figure 2, insets).

As Figure 2 shows, ten explants were put on a single Petri plate (three Petri plates were prepared for a total of three biological replicates for each time point). Over the time-course, swelling of the extremities of the explants was evident from day 6 onwards (Figure 2d) and calli started to appear from day 10 (Figure 2e). At day 20 and 34, the calli increased progressively in mass (Figure 2f,g). Ten explants were pooled per Petri dish to prepare for RNA extraction and subsequent qPCR analysis.

### 2.2. Selection of the Most Representative Time-Points of Nettle Callogenesis via qPCR

A targeted gene expression analysis was performed via qPCR with the goal of identifying the most representative time-points during callogenesis in nettle. The medium used for callogenesis contained a high concentration of auxin (NAA at 3 mg/L) and a lower concentration of cytokinin (BAP at 0.01 mg/L); therefore, primers were designed on a set of genes involved in auxin signaling and transport, as well as cytokinin response. Fifteen genes involved in the metabolism of plant hormones (auxin and cytokinin) and cell cycle were chosen (Table 1), as their expression pattern provides important information on the dynamics of crucial pathways governing plant tissue trans-differentiation.

The genes chosen code for the orthologs of thale cress auxin influx carrier AUX1 [19,20]; the histidine phosphotransfer proteins AHP4-5 (positive regulators of cytokinin signaling [21]); the *Arabidopsis* response regulator (ARR12) protein (also acting in the cytokinin signaling pathway [22]), ARF19, -19-1 and -2B, which are members of the AUXIN RESPONSE FACTOR family [23]; the auxin-responsive proteins IAA21 and IAA22 [24]; the PIN-FORMED auxin efflux carriers located at the plasma membrane, PIN1a and PIN4 [25], and at the endoplasmic reticulum, PIN8 and PIN-LIKES7 (PILS7) [26,27]; and the cyclin D-type proteins CYCD3 and CYCD3.1 (responding to cytokinins and brassinosteroids [28]).

**Figure 3:**
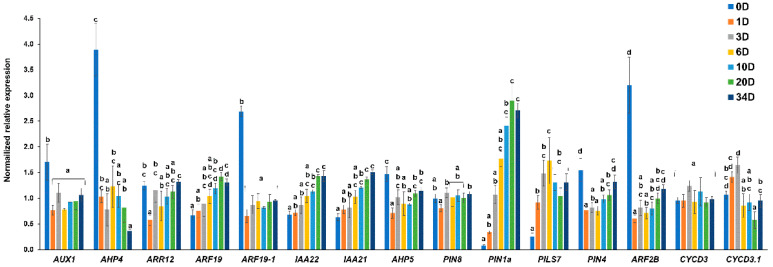
Expression analysis of a set of genes involved in hormone metabolism and cell cycle during callogenesis in nettle explants at 0, 1, 3, 6, 10, 20 and 34 days in the dark. Different letters denote statistical significance among groups obtained via the one-way ANOVA analysis with Tukey’s post hoc test or via the Kruskall–Wallis test with Dunn’s post hoc analysis (*p*-value ≤ 0.05). *AUX1* (F(6,14) = 10.80, *p*-value = 0.000), *PIN8* (F(6,14) = 3.10, *p*-value = 0.038), *PILS7* (F(6,14) = 37.98, *p*-value = 0.000), *PIN4* (F(6,14) = 28.01, *p*-value = 0.000), *CYCD3.1* (F(6,14) = 12.10, *p*-value = 0.000), *AHP4* (*X^2^*(6) = 14.64, *p*-value = 0.022), *ARR12* (*X^2^*(6) = 13.85, *p*-value = 0.031), *ARF19* (*X^2^*(6) = 17.47, *p*-value = 0.008), *ARF19-1* (*X^2^*(6) = 12.58, *p*-value = 0.050), *IAA22* (*X^2^*(6) = 18.04, *p*-value = 0.006), *IAA21* (*X^2^*(6) = 18.42, *p*-value = 0.005), *AHP5* (*X^2^*(6) = 15.37, *p*-value = 0.018), *PIN1a* (*X^2^*(6) = 19.32, *p*-value = 0.004), *ARF2B* (*X^2^*(6) = 17.42, *p*-value = 0.008) and *CYCD3* (*X^2^*(6) = 7.67, *p*-value = 0.263).

The expression analysis showed major changes at the time-points 0, 1, 10 and 34 days (Figure 3). Indeed, as can be seen in Figure 3, the expression patterns can be divided into three major trends: genes drastically decreasing in expression already at day 1 (namely, *AUX1*, *AHP4* and -*5*, *ARR12*, *ARF19-1* and -*2B*, and *PIN4*), increasing with the progression of trans-differentiation (*ARF19*, *IAA21*, *IAA22*, *PIN1a* and *PILS7*), or transcripts that do not show major changes during the kinetics (*PIN8*, *CYCD3* and *CYCD3.1*). As expected, the genes showing the most dramatic response already after 1 day were those coding for the auxin influx carrier AUX1 and the auxin response factors ARF19-1 and -2B, as well as the histidine phosphotransfer proteins AHP4 and -5. However, it should be noted that *ARF19* showed a different trend, since it progressively increased in expression over time. Additionally, the gene coding for the cytokinin-responsive transcription factor ARR12 showed an initial repression at 1 day, followed by an increase as callogenesis progressed. *PIN1a*, which is a member of the PIN-FORMED family controlling lateral root emergence [31], and *PILS7*, a gene controlling auxin signaling [27], increased in expression over time. This finding confirms the similarity existing between the genetic program underlying callogenesis and lateral root formation [16,32]. However, no major variations over time were observed for *PIN8*; an explanation could be ascribed to this gene having a role in processes other than callogenesis in nettle, for example, male gametophyte development, as demonstrated in thale cress [26]. The AUX/IAA genes targeted were progressively induced over time, similarly to what previously shown in thale cress organs undergoing callogenesis using a microarray analysis [32].

From the results obtained with qPCR, the time-points reflecting major transcriptional changes of plant hormone-related genes were 0, 1, 10 and 34 days; indeed, these provided representative information about the progressive increase observed for *ARF19*, *IAA21*, *IAA22* and *PIN1a* and the early decrease characterizing the cytokinin-responsive transcript *AHP4*. Thus, for the subsequent RNA-Seq analysis, these time-points were considered.

### 2.3. Gene Ontology Categories Characterizing Callogenesis at Different Time Points in Nettle

**Figure 4:**
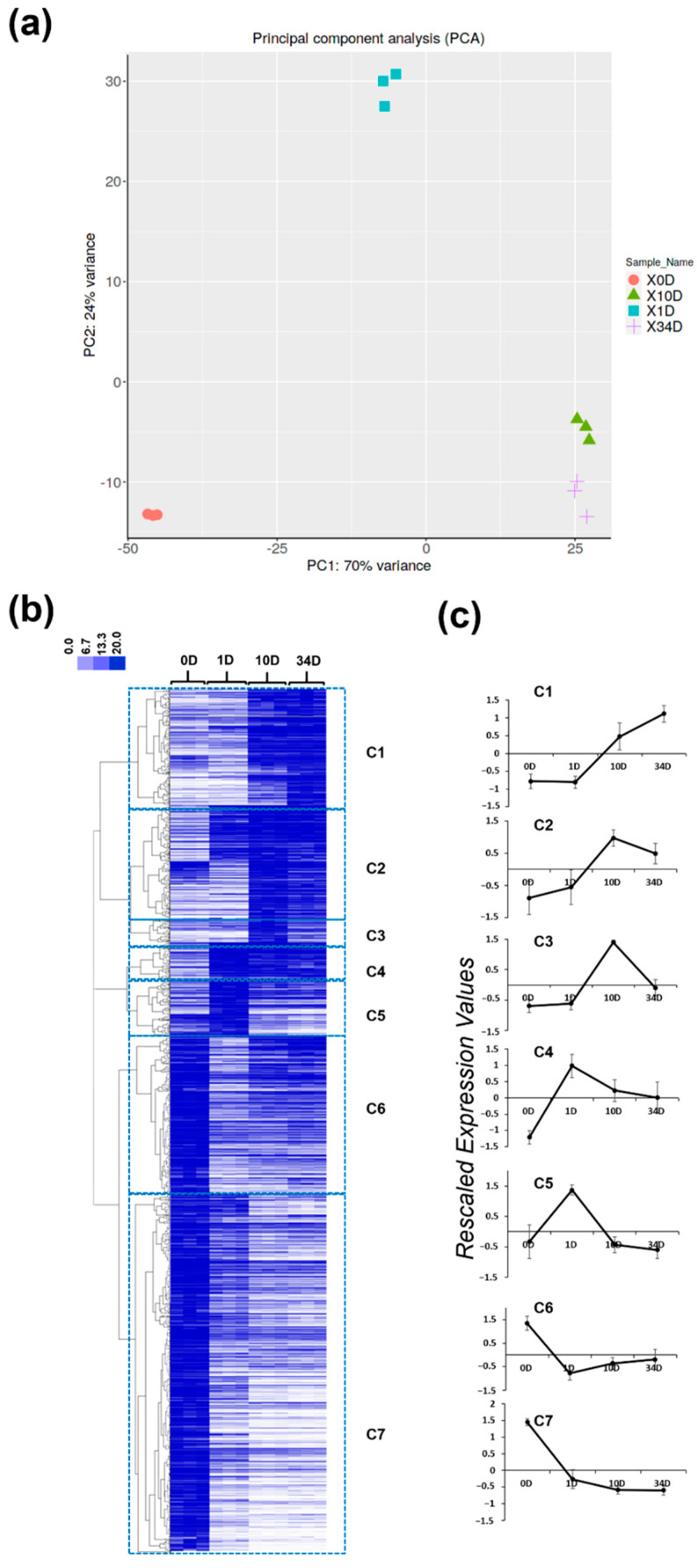
Principal component analysis (PCA) and heatmap of hierarchical clustering with major gene expression profiles. PCA of the RNA-Seq data (**a**). Heatmap of hierarchical clustering of the RNA-Seq data (**b**). C1–C7 clusters obtained using a Pearson correlation coefficient > 0.48 (**c**). The scale bar indicates the expression intensities. (**c**) Profiles of the seven clusters of genes obtained using a Pearson coefficient > 0.48. The profiles are relative to the log2RPKM rescaled values ±  standard deviation (the rescaled values were obtained by subtracting, to the expression value of each contig, the average among the three biological replicates and dividing by the standard deviation).

RNA-Seq data were analyzed with a principal component analysis (PCA) and by performing a hierarchical clustering (HC) of the gene expression data (represented as a heatmap, whereby the intensities of the pixels are proportional to the expression values) with the purpose of identifying major gene expression trends (Figure 4). The PCA highlighted a clear separation between day 0 (0D), 1 (1D) and 10/34 (10D/34D); the two latter time-points clustered indeed closely together (Figure 4a). A pathway analysis on the principal components (PCs) showed that processes related to the generation of precursors/energy, photosynthesis, cell wall polysaccharides and protein-containing subunit organization/assembly characterized PC1 with significant e-values, while PC2 comprised processes related to defense response, the response to external stimuli/stress, wounding and secondary metabolism (Table 2).

The HC analysis revealed the presence of seven clear expression patterns when a Pearson correlation coefficient > 0.48 was selected (Figure 4b). The rescaled expression profiles of each cluster are provided in Figure 4c and are referred to as C1–C7.

C1 is characterized by genes that peaked in expression at 34D; C2 and C3 group transcripts with higher expression at 10D; genes belonging to C4 and C5 increased in expression at 1D, then decreased; C6 and C7 comprise genes expressed at the highest level at 0D.

A gene ontology enrichment (GOE) analysis was performed to obtain an overview of the major ontologies characterizing each of the seven clusters (Dataset S1).

Hereafter, the results are presented and discussed according to the trends highlighted by the clusters.

#### 2.3.1. Ontologies Characterizing the Advanced Stage of Callogenesis (Cluster C1)

##### Iron Deficiency Response

The GOE analysis revealed the presence of ontologies related to the response to reactive oxygen species (ROS) and glutathione metabolic processes (Dataset S1). In the first category, three genes were found: two genes encoding 2-oxoglutarate and ferrous iron Fe^2+^-dependent dioxygenases (contig_3159 and contig_2278, orthologs of *At3g12900* and *At3g13610*) and an alpha dioxygenase (contig_5174, ortholog of *DOX1*-*At3g01420*). These genes all showed a log2FC > 3, when comparing the expression values at 0D vs. 34D, and are highly up-regulated at the last point of the kinetics. In particular, *At3g12900* codes for a scopoletin 8-hydroxylase (S8H) which is involved in the biosynthesis of fraxetin from scopoletin, two coumarins playing a role in strategy I iron uptake in dicots such as thale cress [33,34]. Strategy I relies on the reduction mechanism mediated by the release of protons in the rhizosphere which acidify and increase the solubility of iron in the soil [35].

Together with these genes, an induction of the nettle orthologs of *COSY*-*At1g28680* (contig_153) and *CYP82C4*-*At4g31940* (contig_12259) was also observed (log2FC at 0D vs. 34 D = 1.35 and 1.99, respectively; Dataset S1), a finding pointing to the presence of the complete pathway leading from feruloyl-CoA to sideretin. Here, it is also worth highlighting that the nettle ortholog of the ABC transporter *ABCG37*/*PDR9-At3g53480* (contig_9754) involved in the secretion of scopoletin and derivatives [36] was slightly induced at 34D (the gene is grouped in C6 though), with an FC = 1.6, when comparing 34D vs. 0D (Dataset S1).

COSY, a member of the BAHD acyltransferases, is responsible for lowering the activation energy needed to convert *trans*-*O*-hydroxycinnamoyl-CoA thioesters to *cis*-*O*-hydroxycinnamoyl-CoA thioesters [37], while CYP82C4 is a cytochrome P450 (CYP) involved in the oxidation of fraxetin to sideretin [34]. *CYP82C4*, together with two additional *CYP*s, *CYP71B5* and *CYP82C3*, is induced upon the so-called “iron deficiency response” and it is the only gene correlating with transcripts involved in metal uptake/transport in thale cress [38]. In the RNA-Seq dataset here produced, genes coding for iron transporters were not identified, which, however, does not exclude the possibility that they are up-regulated during callogenesis, given the *de novo* nature of the transcriptome used as reference [9]. In support of the involvement of the iron deficiency response in nettle explants undergoing callogenesis is the up-regulation of a lysyl-tRNA synthetase (contig_30661), ortholog of *AtKRS-1* (*At3g11710*), and of a gene annotated as *B12D* (contig_526), which are among the genes in C1 showing the highest FC difference between 34D and 0D (Dataset S1). The stable expression of the thale cress lysyl-tRNA synthetase in maize triggers translational recoding of lysine into zeins, thereby enriching the lysine content of grains [39]; additionally, *AtKRS-1* is up-regulated in *A. thaliana* under iron deficiency [40]. It was proposed that this gene is involved in the adaptation of plants to stress by promoting translational recoding [39]. In the tomato *chloronerva* mutant, characterized by high apoplastic and symplastic iron concentrations but showing an iron-deficient behaviour, a root-specific lysyl-tRNA synthetase was shown to be up-regulated and to depend on iron availability [41]. Besides its role in protein biosynthesis, plant lysyl-tRNA synthetases may be involved in the synthesis of adenylated 5′-nucleosidyl tetraphosphates (AP4Ns), analogously to what described in *Escherichia coli* [42]. AP4Ns were shown to accumulate in stressed prokaryotic and eukaryotic cells [43,44] and their production in stressed plant cells suggests that they may function as “alarmones” [41,45] (i.e., intracellular signals affecting duplication and gene expression under harsh environmental conditions [46]).

The gene *B12D* codes for an NADH-ubiquinone reductase complex 1 MLRQ subunit and, in rice, its expression was induced upon anoxia, submergence and flooding [47], as well as iron deficiency [48]. This last result corroborates, once more, the presence of the iron deficiency response during nettle callogenesis; the higher expression of nettle *B12D* may be linked to the production of ATP and, hence, to adaptation under high iron requirements (mimicking an iron deficiency syndrome).

Among the transcription factors (TFs) linked to the biosynthesis of coumarins and iron deficiency, it is important to mention *bHLH115* (*At1g51070*) and *WRKY72* (*At5g15130*); the nettle orthologs of these genes (contig_9857 for *bHLH115*; contig_726 and contig_1302 for *WRKY72*) both clustered in C1 and showed a log2FC 34D vs. 0D > 2 and >6, respectively (Dataset S1). The TF bHLH115 is up-regulated in the pericycle upon iron deficiency and interacts with another bHLH, POPEYE, by forming a heterodimer which, in turn, binds BRUTUS, a putative E3 ligase protein functioning as a repressor and coordinating iron homeostasis [49]. WRKY72 was identified as a putative QTL (quantitative trait locus) affecting scopoletin biosynthesis in *A. thaliana* recombinant inbred line populations [50].

##### Scavenging of ROS

Iron deficiency causes an accumulation of other metals, for example, zinc [51], due to the increased activity of ferric-chelate reductase which can accept other metals [41]. An over-accumulation of metals causes, in turn, an increase in ROS production in plant cells and the subsequent need to scavenge them; thus, the lysyl-tRNA synthetase can work in a context of anti-oxidative response. However, what seems a more plausible explanation for the iron deficiency response observed in the present experimental context is the induction of ROS due to tissue trans-differentiation to callus, more than the over-accumulation of other metals; ROS are known to mediate several important physiological responses and developmental stages in plants, among which the maintenance of vegetative apical meristems. The accumulation of ROS leads to the need to scavenge them via antioxidant enzymes, some of which require indeed iron as co-factor (superoxide dismutase, catalase and ascorbate peroxidase [52]). The balance between H_2_O_2_ and O_2_^•^^−^ is, for example, known to be crucial in the root meristem to mark the shift from proliferation to elongation; ROS homeostasis and distribution are regulated via the bHLH transcription factor UPBEAT1-UPB1, which determines the boundaries of the transition zone by negatively regulating peroxidases, thus affecting the concentration of H_2_O_2_ [53]. H_2_O_2_ is scavenged by peroxidases, enzymes known to affect cell wall-related processes in plants by acting in either stiffening or loosening [54]. In the root meristem, their increased activity marks the onset of differentiation. In the experimental dataset here obtained, *UPB1* clustered in C4, together with transcripts highly induced at 1D (Dataset S1). Its expression then decreased, but, at 34D, it was still four-fold higher than that at 0D, a finding indicating that trans-differentiation in nettle is accompanied by mechanisms regulating ROS levels. It remains to be proven whether nettle callogenesis is characterized by a fast increase in H_2_O_2_ levels coinciding with the observed strong up-regulation of *UPB1* at very early stages of trans-differentiation.

Three members of the tau-class of glutathione *S*-transferases [55] were up-regulated in C1, namely, *GSTU7*, -*8* (log2FC 34D vs. 0D > 6) and -*9* (log2FC 34D vs. 0D > 1.4; Dataset S1), a finding confirming the need to activate the antioxidant system in the cells undergoing callogenesis. GSTU7 from thale cress (corresponding to nettle contig_10747 and contig_3454) was shown to antagonize the oxidative stress caused by methyl viologen [56], while *GSTU9* (contig_7301 and contig_4735) was up-regulated in *Arabidopsis* overexpressing a plastidial glycolate oxidase (where H_2_O_2_ is produced in chloroplasts) [57]. Among the TFs induced in C1, it is worth mentioning *TGA2* (contig_10732, log2FC 34D vs. 0D = 1.07), which mediates the response to UV-B and methyl viologen-triggered ROS and induces *GSTU7*, -*8* and -*25* expression by binding to their promoters [58]. The observed induction of *GSTU* genes in nettle at 34D of callogenesis confirms the involvement of ROS in trans-differentiation to callus.

##### Pathogenesis-Related Transcripts

Genes coding for pathogenesis-related proteins (contig_2102, contig_16024 and contig_8710), proteinase inhibitor (contig_2377 and contig_6693), thaumatin-like protein 1 (contig_15054) and an acetone-cyanohydrin lyase (contig_17332) were all induced at 34D (minimum calculated log2FC 34D vs. 0D > 1.5). This is in agreement with what previously shown for thale cress pluripotent calli, where RNA-Seq revealed the induction of several transcripts involved in the response to biotic stress, thus the presence of defense systems against microbial invaders in trans-differentiated cells [59]. The high induction of the acetone-cyanohydrin lyase (also known as hydroxynitrilase) suggests an enzymatic route leading to the liberation of hydrogen cyanide (HCN) from cyanogenic glycosides. These compounds are known to act as “phytoanticipins” (i.e., plant defense compounds acting against biotic agents) [60]; however, a study also suggested that HCN may impact the endogenous levels of ROS and contribute to release dormancy in sunflower seeds [61]. Hence, a signaling pathway linking HCN and ROS homeostasis may exist in nettle calli.

#### 2.3.2. Ontologies Characterizing the Intermediate Stage of Callogenesis (Clusters C2–C3)

##### Immune Response

The GOE analysis of clusters C2 and C3 revealed the presence of transcripts related to the phenylpropanoid pathway, the immune/defense response, salicylic acid (SA) and hypoxia response, and auxin transport (Dataset S1). Among the transcripts linked to the immune response and showing increased expression at 10D, the TF *ERF2* (contig_3897, log2FC 10D vs. 0D > 1.5), which is induced by SA [62], and two genes encoding calmodulin-binding proteins (log2FC 10D vs. 0D > 2.6), namely, *CBP60A* (*At5g62570*, corresponding to contig_9925) and *CBP60G* (contig_10118), were found (Dataset S1). This last gene is a master regulator of immune defense response together with *SARD1* [63], also up-regulated in nettle explants at 10D (contig_3315; Dataset S1). SARD1 does not bind calmodulin, differently from CBP60G, but these TFs activate both the SA-dependent and independent immune response [63]. Both SARD1 and CBP60G can induce the expression of the TF *WRKY70*, whose ortholog in nettle almost doubled in expression at 10D compared to 0D (contig_10625; Dataset S1). WRKY70 is a check-point of the SA- and jasmonic acid (JA)-related stress signals by repressing the latter and stimulating the former [64]. It also acts as either repressor or activator, depending on whether pathogens are present or not; indeed, this TF can bind to the *SARD1* promoter (GACTTTT motif) and repress its expression in the absence of an infection [65].

Calcium–Ca^2+^ signaling is an important aspect of plant defense response. Upon treatment with damage-associated molecular patterns (DAMPs) such as oligogalacturonides, a rapid Ca^2+^ influx was observed in *Nicotiana plumbaginifolia* cell cultures and the use of chemicals blocking the influx failed to activate downstream targets, namely, mitogen-activated protein kinases (MAPK), resulting in the absence of gene activation and the hypersensitive response [66]. Calmodulin-binding proteins, such as those belonging to the CBP60 family, participate in Ca^2+^ signaling and, when induced upon pathogen attack or MAMPs (microbe-associated molecular patterns), can induce the production of SA and, ultimately, the activation of SA-responsive genes [67]. Among the members of CBP60s in thale cress, CBP60A acts as a repressor of the immune response and has a role in conditions where pathogens are not present. When the plant is not facing an infection, *CBP60A* levels are high and those of *CBP60G*-*SARD1* are low, while the reverse happens when a strong MAMP signal is detected [67]. In cluster C2, both *CBP60A* and *CBP60G* are present with the same expression trend peaking at 10D (Dataset S1); since no infection was present, the co-expression of both *CBP60*s may represent a mechanism to control the intensity of the immune response, likely by preventing a full activation, which could exert a negative impact on the proliferation of the callus. In support of the existence of a mechanism controlling immune response is the induction of *MKK6* (*At5g56580*, contig_3282; Dataset S1), an MAPK preventing constitutive activation of immune responses together with *MAPK4* (*At4g01370*) [68]. The nettle ortholog of this gene (contig_3775) is present in cluster 6, but it showed an increased expression (FC > 2.5) at 10D compared to 1D (Dataset S1).

It is also worth mentioning contig_7513, that codes for the ortholog of the MAC perforin domain-containing NSL1 (At1g28380), as well as contig_3372 encoding the ortholog of NUDT7 (At4g12720). Nettle *NSL1* doubled in expression at 10D compared to 0D (Dataset S1); this gene represses MAMP-induced cell death that is triggered by the synthesis of antimicrobial compounds via the production of SA [69]. Nettle *NUDT7* showed a log2FC 10D vs. 0D = 2.6 (Dataset S1); in thale cress, this gene was reported to be a negative regulator of immune response preventing excessive stimulation [70]. Once again, a subtle mechanism regulating immune response via SA-stimulation and controlling its overstimulation exists in nettle explants undergoing callogenesis.

In the experimental set-up here used, rigorous axenic conditions were adopted; therefore, the SA-related transcripts detected are likely linked to an altered cell wall integrity (CWI) status. It is now well established and documented that alterations in the CWI trigger defense responses in plants [71]; receptors strategically located at the interface between cell wall and plasma membrane act as “sentinels” by binding to, e.g., cell wall fragments which act as DAMPs. Several genes related to cell wall modification are indeed present in C4 and C5 as discussed below, a result showing that strong cell wall modifications are induced during callogenesis in nettle explants. This justifies the stimulation of pathways related with SA and, more generally, with the immune response observed at 10D in the absence of an infection. The up-regulation of the wall-associated kinase *WAK2* (contig_15778; Dataset S1) corroborates the hypothesis of alterations in the CWI; this receptor binds to pectins, is induced by SA [72] and is slightly up-regulated following pectin treatment in thale cress protoplasts [73].

Transcriptomics also enabled the identification of another pathway intervening with SA to stimulate the immune response, i.e., the biosynthesis of *N*-hydroxypipecolic acid (NHP). This amino acid derivative intervenes in systemic acquired resistance (SAR), i.e., the long-lasting protection against biotic stressors and its biosynthesis starts with the catalytic action of ALD1 [74] (encoded by *At2g13810*), whose nettle ortholog clusters in C3 and shows a log2FC 10D vs. 0D > 4.5 (contig_17559; Dataset S1). The ortholog of *FMO1* (*At1g19250*, contig_21504) is found in the same cluster and was strongly induced at 10D (Dataset S1). The product of this gene is responsible for the conversion of L-pipecolic acid to NHP [75]. Hence, RNA-Seq indicates that both SA and NHP intervene during nettle callogenesis; in the future, it will be necessary to quantify the levels of these two compounds, to determine their kinetics of accumulation during the proliferation of the callus.

##### Phenylpropanoid Pathway- and Hypoxia-Related Transcripts

It should be noted that several genes intervening in the phenylpropanoid pathway were found in C2 (Dataset S1); orthologs of thale cress *CAD1* and *CAD3* (*At1g72680* and *At2g21890*, corresponding to contig_1059 and contig_4205, respectively, with log2FC 10D vs. 0D > 3.5), as well as *PAL1* (*At2g37040*, contig_4482), which almost doubled in expression at 10D compared to 0D, and two genes coding for dirigent-like proteins (contig_23372 and contig_12966, log2GC 10D vs. 0D > 2.5) were found in the dataset. The phenylpropanoid pathway synthesizes secondary metabolites which play an important role in plant defense response [76].

The intermediate stage of nettle callogenesis is also characterized by the induction of genes related to the response to hypoxia; the nettle ortholog of *HRA1* (*At3g10040*) was found in C2 (contig_5635) and showed a log2FC 10D vs. 0D > 8, together with contig_1034, which is orthologous to *HB1*-*At2g16060* and doubled in expression at 10D compared to 0D (Dataset S1). Pyruvate decarboxylase (contig_472, log2FC 10D vs. 1C > 3.5; Dataset S1), a fermentative enzyme activated upon low oxygen levels [77], was also found in C2. Hypoxia is triggered upon the rapid proliferation of cells typically occurring in the dividing cells of plant calli; the stimulation of cell division triggered by the PGRs auxin and cytokinin triggers an increased consumption of oxygen needed to support the enhanced metabolic demand of the proliferating cell mass, in a manner analogous to that observed in crown galls after *Agrobacterium tumefaciens* infection [78].

##### Transcripts Involved in the Metabolism of Brassinosteroids and Auxins

Ontologies related to lipid biosynthesis and, more specifically, to the metabolism of brassinosteroids (BRs) were also identified at the intermediate stage of callogenesis. The orthologs of *MEE31* (*At3g02570*, contig_6483, log2FC 10D vs. 0D = 1.1), *BAS1* (*At2g26710*, contig_10801, log2FC 10D vs. 0D > 5), *CYP716A1* (*At5g36110*, contig_5745, log2FC 10D vs. 0D > 2.5) and *CYP724A1* (*At5g14400*, contig_29844, log2FC10D vs. 0D = 6) were upregulated at 10D (Dataset S1). The involvement of BR-related genes likely depends on an alteration in the circadian rhythm. Callogenesis was set up in complete darkness; therefore, the differential regulation observed in these genes may be due to the experimental condition chosen.

*MEE31*, also known as *PMI1* (phosphomannose isomerase 1), displays diurnal variations in expression and, more specifically, is induced in the light, upon an increase in ascorbic acid levels; MEE31 was also shown to be responsible for ascorbic acid synthesis in thale cress from hexose phosphates via the D-Man/L-Gal pathway [79]. The other genes of the same pathway are also known to respond to the light-dark cycle; for example, *BAS1*, encoding a cytochrome P450, showed a log2FC 10D vs. 0D = 5.1 (Dataset S1) and is a BR catabolic gene catalyzing the hydroxylation of brassinolide to the inactive form 26-hydroxybrassinolide [80]. Thus, during callogenesis, genes involved in BR signaling intervene in the adaptation to the prolonged dark conditions of callogenesis and transcripts regulating the conversion of active forms of the PGR to inactive ones may play a role in keeping the right concentrations of active forms. However, this needs validation via quantification of BRs.

Auxin-related genes were also found in C2; *ABCB4* (*At2g47000*, contig_9223), *AGG2* (*At3g22942*, contig_12241) and *ZIFL1* (*At5g13750*, contig_11950) all showed a log2FC10D vs. 0D > 2 (Dataset S1). As discussed above, in Section 2.2, auxin-related genes were expected to vary significantly in expression given the use of the PGR in the medium. An additional piece of information is provided by the expression pattern of *AGG2*; this gene acts by increasing the stability of *NDL1*, required for local auxin maxima and is induced by high auxin levels [81]. Here, it is relevant to mention that the expression of *NDL1* decreased with callogenesis (the gene was found in C7) and that a feedback signaling mechanism controlling auxin transport and gradient via the action of *AGG2*–*NDL1* exists in nettle explants during callogenesis.

#### 2.3.3. Ontologies Characterizing the Early Stages of Callogenesis (Clusters C4, C5, C6 and C7)

##### Transcripts Involved in Cell Wall Loosening

The early stage of callogenesis (1D) is characterized by ontologies related with cell wall catabolic processes, cell wall loosening and metabolism of dicarboxylic acids and cytokinins (Dataset S1). Already 24 h after exposure of the explants to the PGRs, cell wall remodeling-related processes were induced. Three members of the glycosyl hydrolase family 9B (GH9B) were indeed found in C4, i.e., *GH9B1*-2-*6*-*8* (*At1g70710*, *At1g02800*, *At1g23210* and *At2g32990*, corresponding to contig_29909, contig_29728, contig_22184 and contig_19509, respectively, all with log2FC 1D vs. 0D > 2; Dataset S1). GH9s belong to the CAZY (Carbohydrate-Active enZYmes Database; http://www.cazy.org/, accessed on 13 September 2021) family of inverting enzymes formerly known as cellulase family E and grouping also endoglucanases; the enzymes belonging to this family have been classified into three groups, namely A, B and C, depending on whether they have a transmembrane domain, a signal peptide, or a signal peptide with a carbohydrate binding module CBM49 [82].

*GH9B1* (also known as *cel1*) is expressed in expanding tissues of thale cress and accumulates particularly in xylem cells [83] and its knock-down triggers shorter stems and roots, as well as less lignified xylem and phloem cells [84]. The expansins identified, *EXPA1,* -*4 and* -*8* (*At1g69530*, *At2g39700* and *At2g40610*, corresponding to contig_33240/contig_20613/contig_34011/contig_32221, contig_9569 and contig_31689/contig_35761, all with a log2FC 1D vs. 0D > 1.8), as well as the genes involved in pectin catabolism (*At3g07010*, *At4g13710*, *At5g63180* and *At4g33220*-*PME44*, corresponding to contig_29771, contig_28278, contig_11050 and contig_27620, all with a 0.4 < log2FC 1D vs. 0D = 4.5) and *MIOX4* (*At4g26260*, contig_5715 with log2FC 1D vs. 0D > 3) (Dataset S1) indicate major cell wall remodeling processes accompanying nettle callus formation. The presence of auxin in the medium is responsible for the major transcriptional changes observed, since this PGR is known to induce cell wall loosening [85]. Auxin induces acid growth via activation of H^+^-ATPase proton pumps [86,87] and the acidic pH stimulates the activity of expansins [88]. Expansins loosen the connections between cellulose microfibrils and non-cellulosic polysaccharides making the former slide apart, thereby favoring relaxation of the walls. The low pH also stimulates the activity of pectin-related genes, namely, pectin methylesterases (PMEs) which de-methylesterify homogalacturonans either in a block-wise or random manner, ultimately affecting the mechanical properties of the cell wall (reviewed in [89]). The induction of two *PME*s, together with several pectin lyases in C4–C5 suggests the likely occurrence of random de-methylesterification; randomly de-methylesterified pectins are substrates of polygalacturonases and pectin lyases (increasing cell wall loosening), while block-wise de-methylesterified pectins give rise to a gel via the interaction with Ca^2+^ [90].

Among the cell wall-related genes induced at 1D, it is worth mentioning *MIOX4*, coding for a *myo*-inositol oxygenase; this enzyme is implicated in the synthesis of cell wall nucleotide sugar precursors, more specifically UDP-GlcA (glucuronic acid), as an alternative pathway to the direct oxidation of UDP-glucose [91]. However, *MIOX4* was also shown to be involved in ascorbic acid biosynthesis by means of over-expression studies [92], via the conversion to L-gulonate, although subsequent radiotracer experiments in *Arabidopsis* using a frame-shift mutant of glucuronokinase 1 did not show any accumulation of labelled ascorbic acid [93]. It must be said that, in the present study, no unequivocal conclusions can be drawn as to whether *MIOX4* is the preferred route for UDP-GlcA biosynthesis during the early stage of nettle callogenesis, since data on the differential expression of genes involved in the synthesis of *myo*-inositol are not present and the quantification of UDP-xylose, known to inhibit UDP-glucose dehydrogenase via a negative feed-back [94], was not performed.

Among the genes induced at 1D, three transcripts encoding DUF642 family proteins were found: contig_29087, contig_12994 and contig_21054 (Dataset S1). DUF642 proteins are generally related to changes in pectin methyl-esterase activity or changes to the degree of methyl-esterification of homogalacturonans [95]; for example, the DUF642 genes *At4g32460* and *At5g11420* had a positive effect on seed germination when overexpressed in thale cress, via promotion of PME activity and testa rupture [96] and *At3g08030* was proposed as a marker of seed ageing, as its expression was positively correlated with seed germination [97].

##### Transcripts Related with the Metabolism of Organic Acids and Cytokinins

In the early phase of callogenesis, an induction of transcripts involved in the metabolism of dicarboxylic acids was also observed (Dataset S1). Organic acids such as malate and fumarate were shown to act as temporary C-storage in C3 plants, such as *A. thaliana* after a prolonged dark period, to sustain respiration after carbohydrate depletion [98]. The darkness imposed in the experimental set-up here chosen triggered clear and needed metabolic adjustments to support the metabolism under the prolonged absence of light.

A fine balance of active cytokinins, at least at the transcriptomic level, was also present during early callogenesis; three cytokinin metabolism-related genes, *CKX1* and -*6* (*At2g41510* and *At3g63440*, corresponding to contig_13913 and contig_12745, both with a log2FC 1D vs. 0D > 2.5) and *LOG7* (*At5g06300*, corresponding to contig_32442, log2FC 1D vs. 0D = 7.9) were identified (Dataset S1). *CKX*s encode cytokinin oxidases [99] catalyzing the degradation of the PGR, while *LOG7* codes for a cytokinin nucleoside-monophosphate phosphoribohydrolase releasing active cytokinins [100]. An increased expression of some *CKX*s was previously also observed in *Arabidopsis* explants cultivated on a callus-inducing medium containing kinetin [32], while *LOG7* was up-regulated in wounded explants showing callus formation [17]. Therefore, the balanced action of exogenous and endogenous cytokinin triggered upon wounding of the stem internode explants likely coordinate the early phase of nettle callogenesis.

##### Transcripts Involved in Base Excision Repair, Cell Wall Biosynthesis and Photosynthesis

Several gene ontologies were found in nettle internode explants transferred on a callus-inducing medium at the beginning of callogenesis (0D) and a comparison between the extreme time-points, i.e., 34D vs. 0D, revealed 259 transcripts up-regulated at 0D (Dataset S1).

Here, the results are discussed by focusing on three crucial pathways, given their documented role in the determination of plant cell fate [101,102] and because it would be too broad to treat in detail all the gene ontologies enriched at 0D. Here, the focus is given to base excision repair- and cell wall-related processes, as well as photosynthesis-related ontologies.

Base excision repair (BER) is a mechanism regulating genome defense by repairing lesions to DNA caused by genotoxic agents; it is also responsible for another important pathway, namely, the replacement of 5-methylcytosine with cytosine in active DNA demethylation [103]. Callogenesis requires changes in the chromatin structure of plant cells; it was indeed demonstrated that, during the leaf-to-callus transition in thale cress, changes in DNA methylation occurred, with CHG regions showing increased methylation and CHH demethylation [104]. Epigenetic reprogramming linked to chromatin configuration is involved in callogenesis; to erase leaf identity genes, trimethylation of histone 3 lysine 27 (H3K27me3) occurs during the leaf-to-callus transition [105].

In the present RNA-Seq dataset, three genes involved in BER were up-regulated at 0D (0.53 ≤ log2FC 0D vs. 1D ≤ 2.68; Dataset S1) and progressively down-regulated as callogenesis progressed—contig_31442 and contig_22351 (corresponding to the DNA glycosylases *At3g12710* and *At5g44680*) and contig_22270 (corresponding to *At2g36490*-*DML1*, a DNA demethylase involved in *Fusarium* resistance in *Arabidopsis* [106]). The explants used for the present study derive from the stem and the observed down-regulation of genes involved in BER can be explained by the need to repress genes involved in establishing the identity of aerial organs and favor callogenesis.

Three genes involved in secondary cell wall biosynthesis, namely, *IRX1*, *IRX3* and *IRX12* (corresponding to contig_27738, contig_22306 and contig_31490, respectively) also showed a progressive down-regulation with the progress of callogenesis (2.50 ≤ log2FC 0D vs. 1D ≤ 3.08; Dataset S1). *IRX1* and *IRX3* code for two cellulose synthases involved in secondary cell wall biosynthesis, i.e., CESA8 and CESA7 [107]; their down-regulation accompanied the loosening observed at 1D and confirms the important contribution of cell walls to the identity of plant cells. For example, the modification or complete removal of the cell wall (to generate protoplasts) is known to be a component of in vitro trans-differentiation and pluripotency [108,109].

Callogenesis was performed under dark conditions; thus, to observe a number of differentially regulated photosynthesis-related transcripts was expected. Among them, genes involved in chloroplast relocation showed a statistically significant down-regulation at 1D compared to 0D; phototropins (corresponding to contig_27228 and contig_14966), as well as the glutaredoxin family protein AT1G64500 involved in actin bundling and chloroplast movement (corresponding to contig_2269) displayed a log2FC 0D vs. 1D > 1.5 (Dataset S1). The changes triggered on the photosynthetic machinery accompany the switch to a heterotrophic metabolism, an alteration of the source/sink balance and the subsequent loss of photosynthetic capacity and re-entry into the cell cycle. Mechanically separated *Asparagus* cells showed a loss of photosynthetic capacity associated with the onset of the cell cycle and accompanying chloroplast trans-differentiation to proplastid-like structures [110].

## 3. Materials and Methods

### 3.1. Plant Materials and Growth Conditions

Terminal shoots containing several nodes were collected from healthy vigorous nettle plants (i.e., the fibre clone 13) growing in the incubators under a cycle of 16 h light at 25 °C and 8 h dark at 20 °C, as previously reported [111]. The shoots were first washed with detergent (i.e., water with a few drops of soap) for 10 min, then submerged in 50% (*v*/*v*) commercial bleach for 10 min, followed by three rinses with sterilized distilled water. Segments bearing a single node of the surface-sterilized shoots were put vertically onto Murashige and Skoog (MS) basal medium containing vitamins (Duchefa Biochemie, Haarlem, The Netherlands), 3% (*w*/*v*) sucrose and 0.8% (*w*/*v*) agar and grown in a culture room under light-emitting diode (LED) lights (Philips GreenPower LED production module DeepRed/Blue) with a 16/8 h light/dark cycle at a constant temperature of 22 °C. The obtained in vitro plantlets were sub-cultured by transferring terminal shoots to the same fresh media. The internode explants of 1 cm length were excised from 4-week-old plantlets and used for the subsequent experiments.

### 3.2. Optimal Concentrations and Combinations of Plant Growth Regulators for Callus Induction

To determine the optimal concentration of BAP and NAA for callus induction, the combination of different concentrations was first examined using 100 mm square Petri dishes with 25 (5 × 5) compartments (Thermo Fisher Scientific, Bremen, Germany). Various concentrations of both BAP and NAA (3, 1, 0.5, 0.1 and 0.01 mg/L) were used to obtain a total of 25 combinations (Supplementary Figure S1). These BAP–NAA combinations were added to the MS basal medium supplemented with vitamins, 0.1% (*w*/*v*) KNO_3_, 0.08% (*w*/*v*) NH_4_NO_3_, 0.12% (*w*/*v*) KH_2_PO_4_, 1% (*w*/*v*) sucrose and 0.3% (*w*/*v*) Gelrite (Duchefa Biochemie, Haarlem, The Netherlands). The internode explants were placed horizontally onto the medium and cultured in the dark or under the same condition as in vitro plants (two Petri dishes per condition).

### 3.3. Sampling, RNA Extraction, qPCR and Statistics

The internode explants were cultured on the medium supplemented with 3 mg/L of NAA and 0.01 mg/L of BAP in the dark and collected at 0, 1, 3, 6, 10, 20 and 34 days. Ten explants from the same plate were pooled to generate one biological replicate for each time point and three biological replicates were collected. Total RNA extraction was performed using the RNeasy Plant Mini kit (Qiagen, Leusden, The Netherlands) according to the manufacturer’s instructions. RNA quantity was measured using a NanoDrop ND-1000 spectrophotometer (Thermo Fisher Scientific, Villebon-sur-Yvette, France). RNA quality was assessed with a 2100 bioanalyzer (Agilent, Santa Clara, CA, USA). cDNA synthesis was carried out using one µg of total RNA and ProtoScript II RTase (NEB) according to the manufacturer’s instructions. All primers were designed with the online program Primer3Plus (http://www.bioinformatics.nl/cgi-bin/primer3plus/primer3plus.cgi/ (accessed on 26 July 2019) and primer efficiency was calculated using 5-fold serial dilutions of cDNA ranging from 0.0008 ng/µL to 12.5 ng/µL. Target genes were identified by blasting thale cress orthologs in the nettle leaf database at Blast4OneKP (available online at http://db.cngb.org/blast4onekp/home (accessed on 26 July 2019); [29,30]). Primer sequences and amplification efficiencies are provided in Table 1. Five reference genes reported previously [111] were used in this study. Of these five, two reference genes (i.e., *RAN* and *eTIF4E*) were selected to calculate the relative gene expression, as they were determined to be the most stable by geNorm implemented in the qbase+ software v3.2 (Biogazelle, Zwijnaarde, Belgium). The normality and homogeneity of the data were checked using a Shapiro–Wilk test and a Levene’s test, respectively. A one-way ANOVA with a Tukey’s post hoc test was used when parametric testing was possible, while a Kruskal–Wallis with Dunn’s post hoc test was used when parametric test’s conditions were not met. The statistical analyses were performed with IBM SPSS Statistics v20 (IBM SPSS, Chicago, IL, USA).

### 3.4. Preparation of the Libraries, RNA-Seq Analysis and Validation with qPCR

The SMARTER stranded RNA-Seq Kit was used to prepare twelve libraries (i.e., three biological replicates for samples collected at 0, 1, 10 and 34 days) following the manufacturer’s instructions (Takara Bio, Saint-Germain-en-Laye, France) and as previously described [9]. The libraries were pooled and sequenced to generate 75 bp paired-end reads using an Illumina NextSeq500 instrument with the NextSeq500/550 Mid Output Kit v2.5 (150 cycles; Illumina, San Diego, CA, USA). Raw sequences were deposited in NCBI Gene Expression Omnibus (GEO) with the accession number GSE185155. The raw sequences were uploaded to CLC Genomics Workbench v. 11.0.1 (QIAGEN Aarhus A/S, Denmark). The sequences were treated as previously described [9]: sequences > 35 bps were retained, the sequence quality score was, by default, 0.05 and the maximum number of ambiguities was set to 0. Adaptor trimming was performed using the Illumina adaptor sequences, then a hard trim of 15 bps at the 5′ end and 3 bps at the 3′ end was additionally carried out, resulting in a final sequence average length of 57 bps. Mapping was performed against the previously published de novo transcriptome of nettle clone 13 [9] with the following parameters: maximum hit per read = 3; similarity and length fraction = 0.95; mismatch/insertion/deletion cost = 3. The expression values were calculated using the RPKM (reads per kilobase of transcript per million reads mapped) method [112]. An ANOVA one-way statistical test with four groups (0, 1, 10 and 34 days) was applied, each composed of 3 biological replicates; thereafter, a false discovery rate (FDR) correction was applied. Only those genes showing a corrected *p*-value < 0.05 were retained for the bioinformatics analysis. The data were filtered by removing the genes showing a maximum value of the means < 1 RPKM and a maximum FC > |4|. A total of 3143 contigs were obtained (Dataset S1). The Gene Ontology term enrichment (GOE) analysis was performed using Cytoscape v3.7.1 [113] with the ClueGO v2.3.2 plugin [114] (*p*-value < 0.05; Benjamini–Hochberg enrichment, gene ontology from level 3 to level 8; kappa score = 0.4). Principal component analysis (PCA) and PCA pathway analysis were carried out using iDEP.93 [115]. Gene expression patterns were hierarchically clustered using Cluster 3.0 [116] and plotted as a heatmap with Java Treeview [117] (available online at http://jtreeview.sourceforge.net/ (accessed on 1 September 2020)).

For qPCR validation, the following contigs were chosen: contig_9978, contig_11349, contig_27738, contig_31490, contig_35897, contig_4597, contig_25850 and contig_7440. The primers used have been previously reported [9]. The expression values were calculated with qbase+ as described in Section 3.3 and the reference genes used were *RAN* and *eTIF4E* [111]. The log2 fold change (FC) of the NRQs (normalized relative quantities) and RPKM were plotted and the R^2^ calculated (Dataset S1).

## 4. Conclusions

The results obtained in this study provide a comprehensive view of the transcriptional changes accompanying callogenesis in nettle stem explants. Key gene ontologies dominate at the different time-points studied and several new findings have been provided. Among the new findings, there is the iron deficiency response at the advanced stage of callogenesis triggered by the ROS and the need to scavenge them via antioxidant enzymes relying on iron as a co-factor. The up-regulation of genes related to the immune response at the intermediate stage is likely caused by an altered cell wall status, as evidenced by the major changes observed in cellulose- and pectin-related processes. The RNA-Seq data here provided will be a useful resource for researches focusing on plant callogenesis and on nettle-related applications based on tissue culture and bioprocess engineering.

## Figures and Tables

**Table 1:** Details of the genes and qPCR primers used in this study with indication of the sequence reference (contig/scaffold number relative to the previously published *de novo* assembly [9], or to the scaffold number from the onekp database [29,30]), respective gene names and amplification efficiencies (%).

Sequence Reference	Gene	Forward Primer (5′→3′)	Reverse Primer (5′→3′)	Efficiency %
contig_19482	*AHP4*	TTCGTGGAGGAAATCGCTAC	CTTTCAGCTGGTGCATGAAG	109.9
WKCY_scaffold_2048943	*AHP5*	CGAGGCTCAGAATTTGGAAG	GCTCAAGCATGAACAGTTGG	99.6
contig_1005	*ARF2B*	ATGAGGGCGATATGATGCTC	GTCTCCATGCGAATTTAGGG	100.3
contig_3166	*ARF19*	CTGAAGCGTCATCTGAGCATAC	CGTCCATGAACATGAACACC	90.6
WKCY_scaffold_2007870	*ARF19-1*	TTGCATGCCGATACAGAGAC	AAAACTCGGTGGGTTGTCTG	101.3
contig_1723	*ARR12*	TGCTCTTCGTCCTCTTTTCC	ATGGGTTTCATCGCAGTCTC	97.3
contig_2471	*AUX1*	CGAATGCCCAATACACACAG	GAGATTATGCACGCGATGTG	92.0
contig_3855	*IAA22*	TTTTGGTGGCCGAAGTAGTC	CTCCGTGTTCAAGCATTTCC	100.5
contig_15219	*IAA21*	TCGTTCTCCTGAACATGCTG	TGTCCACCTCCCTTTTTCAG	89.5
WKCY_scaffold_2013249	*CYCD3*	TTGCACTGGGAGTTTCTGTG	TGATCACGAGGAGCATTGTC	94.7
contig_25826	*CYCD3.1*	TCTTGCTGGAACAAGACCTG	ATGCTCTTCGTCTTCCTTGG	92.1
contig_20694	*PIN8*	CAGGCTGTGATGCGTAATTC	GAACCAGCAATGTTCGATCC	97.2
contig_29151/WKCY_scaffold_2010532	*PIN1a*	TGGGATGGCCATGTTTAGTC	AAACAACGCCACTGAGTTCC	95.2
contig_8278	*PIN4*	TCTCCAGAAGCTCATCATGC	TGGAGAGGGAGAAGATGGTG	95.8
contig_5072	*PILS7*	ACGCTGTTTGAAGTGGCATC	CGAAGTTGCCAAGAAAGCTC	99.7

**Table 2:** Pathway analysis on PC1 and PC2 of the PCA shown in Figure 4a. The e-values of each pathway are indicated.

Component	Pathways	e-Value
**PC1**	Generation of precursor metabolites and energy	1 × 10^−^^2^
	Cell wall polysaccharide metabolic process	5 × 10^−^^2^
	Photosynthesis	2 × 10^−^^12^
	Photosynthesis, light reaction	1 × 10^−^^5^
	Protein-containing complex subunit organization	2 × 10^−^^2^
	Protein-containing complex assembly	2 × 10^−^^2^
**PC2**	Regulation of response to biotic stimulus	5 × 10^−^^3^
	Defense reponse	8 × 10^−^^3^
	Response to wounding	5 × 10^−^^3^
	Secondary metabolic process	9 × 10^−^^3^
	Regulation of response to external stimulus	2 × 10^−^^3^
	Regulation of response to stress	8 × 10^−^^3^

## Data Availability

Raw FASTA files, as well as processed RNA-Seq data, were deposited in GEO (GSE185155). The processed RNA-Seq data are included in Dataset S1.

## Supplementary Materials

The following are available online at https://www.mdpi.com/article/10.3390/ijms222212319/s1.
