# Supplementary material for: Transcriptomic Changes in Internode Explants of Stinging Nettle during Callogenesis
Source: Int J Mol Sci. 2021 Nov 15;22(22):12319. doi: 10.3390/ijms222212319 (PMC8618292; doi:10.3390/ijms222212319)
Supplement: Supplementary file 1 [file ijms-22-12319-s001.zip › ijms-1452018-supplementary.pdf]

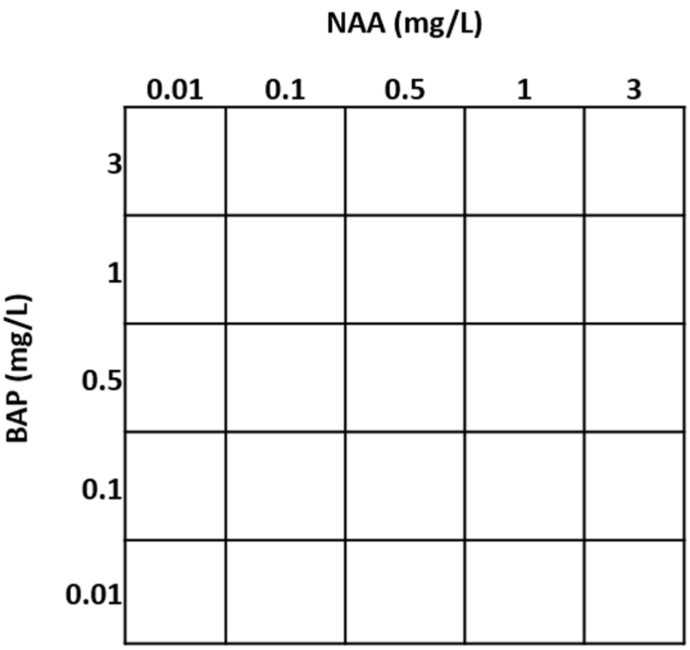

Figure S1. Scheme showing the combination of different concentrations of BAP and NAA.
